# Lymphocyte Subsets Show Different Response Patterns to *In Vivo* Bound Natalizumab—A Flow Cytometric Study on Patients with Multiple Sclerosis

**DOI:** 10.1371/journal.pone.0031784

**Published:** 2012-02-20

**Authors:** Andrea Harrer, Georg Pilz, Max Einhaeupl, Katrin Oppermann, Wolfgang Hitzl, Peter Wipfler, Johann Sellner, Stefan Golaszewski, Shahrzad Afazel, Elisabeth Haschke-Becher, Eugen Trinka, Joerg Kraus

**Affiliations:** 1 Department of Neurology, Christian-Doppler-Klinik, Paracelsus Medical University, Salzburg, Austria; 2 Central Laboratory, Christian-Doppler-Klinik, Paracelsus Medical University, Salzburg, Austria; 3 Paracelsus Medical University, Research Office, Biostatistics, Salzburg, Austria; Institute Biomedical Research August Pi Sunyer (IDIBAPS) - Hospital Clinic of Barcelona, Spain

## Abstract

Natalizumab is an effective monoclonal antibody therapy for the treatment of relapsing- remitting multiple sclerosis (RRMS) and interferes with immune cell migration into the central nervous system by blocking the α_4_ subunit of very-late activation antigen-4 (VLA-4). Although well tolerated and very effective, some patients still suffer from relapses in spite of natalizumab therapy or from unwanted side effects like progressive multifocal leukoencephalopathy (PML). In search of a routine-qualified biomarker on the effectiveness of natalizumab therapy we applied flow cytometry and analyzed natalizumab binding to α_4_ and α_4_ integrin surface levels on T-cells, B-cells, natural killer (NK) cells, and NKT cells from 26 RRMS patients under up to 72 weeks of therapy. Four-weekly infusions of natalizumab resulted in a significant and sustained increase of lymphocyte-bound natalizumab (p<0.001) which was paralleled by a significant decrease in detectability of the α_4_ integrin subunit on all lymphocyte subsets (p<0.001). We observed pronounced natalizumab accumulations on T and B cells at single measurements in all patients who reported clinical disease activity (n = 4). The natalizumab binding capacity of *in vitro* saturated lymphocytes collected during therapy was strongly diminished compared to treatment-naive cells indicating a therapy-induced reduction of α_4_. Summing up, this pilot study shows that flow cytometry is a useful method to monitor natalizumab binding to lymphocytes from RRMS patients under therapy. Investigating natalizumab binding provides an opportunity to evaluate the molecular level of effectiveness of natalizumab therapy in individual patients. In combination with natalizumab saturation experiments, it possibly even provides a means of studying the feasability of patient-tailored infusion intervals. A routine-qualified biomarker on the basis of individual natalizumab saturation on lymphocyte subsets might be an effective tool to improve treatment safety.

## Introduction

Recruitment of activated immune cells across the blood-brain barrier (BBB) into the central nervous system (CNS) is considered essential for the initiation of inflammatory brain lesions in multiple sclerosis (MS) [1], [2]. Integrins are shown to be major players of immune cell trafficking. The two most relevant interactions of immune cell transmigration across the BBB involve firm adhesion of the vascular cell adhesion molecule-1 (VCAM-1) on endothelial cells with very late activation antigen-4 (VLA-4, α_4_β_1_ integrin) on leukocytes and of the endothelial intercellular adhesion molecule-1 (ICAM-1) with leukocyte function associated antigen-1 (LFA-1, α_L_β_2_ integrin) on immune cells [3], [4]. The pathogenic relevance of the α_4_β_1_ integrin was identified as early as 1992 by Yednock et al. who demonstrated that CNS accumulations of leukocytes in experimental autoimmune encephalomyelitis (EAE) are effectively prevented by treatment with antibodies specific for the α_4_ subunit [5].

Natalizumab was designed on the basis of mouse progenitor antibodies by engineering complementarity-determining regions onto a human IgG4 framework. In contrast to other IgG subclasses, IgG4-antibodies are mere blocking antibodies with minor affinity to immune cell Fc receptors, and they do not bind complement. Hence, they are neither involved in antibody-dependent cell-mediated nor in complement-dependent cellular cytotoxicity [6]. Natalizumab blocks immune cell extravasation into the CNS by selectively binding to the α_4_ subunit of VLA-4 [7]. It is the first monoclonal antibody therapy approved for treatment of MS and was shown to impressively reduce relapse frequency and disease progression in patients with relapsing-remitting MS (RRMS) [8], [9]. However, the overall dimension of the pharmacological activity of natalizumab is unsolved and clinical effectiveness is counteracted by the risk to develop progressive multifocal leukoencephalopathy (PML).

Current knowledge on further mechanisms of action include an increase of leukocyte counts, nucleated erythrocytes, pre-B cells, and CD34+ hematopoietic stem cells [10], [8], [11] in the peripheral blood, reduced serum levels of soluble VCAM-1 [12], a sustained decrease in immune cell numbers in the cerebrospinal fluid [13], and depletion of dendritic cells in cerebral perivascular spaces [14]. Notably, the elevation of peripheral CD34+ cells and pre-B cells might have pathogenic relevance for the development of PML since the bone marrow was identified as reservoir of the JC virus [15], [16]. According to the latest global natalizumab safety update 201 cases of PML have been reported through Jannuary 4^th^, 2012 (www.fda.gov/Drugs/DrugSafety/ucm288186.htm). The risk of PML apparently is time-dependent and the median therapy duration to onset of PML symptoms was reported to be 25 months [17].

Occurrence of neutralizing anti-natalizumab antibodies (NAB) is another important phenomenon influencing therapeutic effectiveness. NAB have been detected in 9% of natalizumab-treated patients from the AFFIRM study. Two thirds (6%) thereof remained persistently NAB-positive and exhibited a reduced clinical efficacy [18]. A more recent study reported persistent NAB in 3.5% and non-persistent NAB in 1% of 4881 patients suggesting lower frequencies of NAB [19].

Continuous efforts are required to early differentiate patients with ongoing disease activity from patients with a solid treatment response and to identify patients at risk to develop PML. Flow cytometry might represent an appropriate method to investigate individual treatment responsiveness by analyzing surface levels of *in vivo* bound natalizumab.

## Materials and Methods

### Participants

Twenty-six patients (23 females, 3 males; mean age 35.1 years ±9.3) with clinically definite RRMS according to the revised McDonald criteria [20] were included in the study (Table 1). A relapse was defined as acute or subacute onset of objective symptoms of neurological disturbances that lasted for more than 24 hours and could be attributed to MS. Pseudoattacks were excluded. Patients were recruited from the Department of Neurology, Paracelsus Medical University, Salzburg, and received natalizumab according to the European Medicines Agency's indication due to active disease despite previous immunomodulatory therapy. Steroid therapy less than one week ago and ongoing infections were exclusion criteria for natalizumab infusions. Patients received infusions of the standard 300 mg dose of natalizumab every four weeks. Routine examinations for NAB were performed before the second, third, and seventh natalizumab infusion. Peripheral venous blood was collected before initiation of natalizumab therapy (T0, n = 26), after 12 weeks (T12, n = 26), 24 weeks (T24, n = 18), 36 weeks (T36, n = 18), 48 weeks (T48, n = 18), and 72 weeks (T72, n = 10) prior to subsequent infusions. The study was approved by the local ethics committee (Ethikkommission für das Bundesland Salzburg 415-E774/6-2007) and all participants gave written informed consent.

**Table 1:** Patient Characteristics - Clinical Parameters.

		Age (y)			During natalizumab Therapy					Previous Therapies
ID	Sex	Onset^1^	T0	Relapses^2^	EDSS T0	Relapses^3^	Last EDSS	Duration (w)	NAB	
1	F	28	31	2	4.5	**2**	4.0	36	**positive** 4	22 µg IFN-ß-1a (s.c.), GA
2	F	28	29	4	0.0	0	1.0	48	negative	22 µg IFN-ß-1a (s.c.), 44 µg IFN-ß-1a (s.c.)
3	M	29	53	2	2.5	0	2.5	72	negative	IVIG, IFN-ß-1b (s.c.)
4	F	18	24	2	0.0	0	0.0	48	negative	22 µg IFN-ß-1a (s.c.), 44 µg IFN-ß-1a (s.c.), IFN-ß-1a (i.m.), GA
5	F	21	25	6	1.0	0	1.0	72	negative	44 µg IFN-ß-1a (s.c.)
6	F	36	41	1	1.5	0	1.5	72	negative	GA, 22 µg IFN-ß-1a (s.c.), 44 µg IFN-ß-1a (s.c.), Mitoxantrone
7	F	48	50	4	3.0	**1**	3.0	72	negative	22 µg IFN-ß-1a (s.c.), 44 µg IFN-ß-1a (s.c.)
8	F	23	33	4	3.0	0	2.5	48	negative	22 µg IFN-ß-1a (s.c.), 44 µg IFN-ß-1a (s.c.)
9	F	38	39	3	4.0	0	4.0	48	negative	22 µg IFN-ß-1a (s.c.), 44 µg IFN-ß-1a (s.c.)
10	F	33	35	2	3.0	**2**	4.5	72	negative	22 µg IFN-ß-1a (s.c.), 44 µg IFN-ß-1a (s.c.)
11	F	25	47	2	5.0	0	4.5	48	negative	IFN-ß-1a (i.m.), GA
12	F	23	28	1	4.5	0	4.5	48	negative	IFN-ß-1a (i.m.), 9.6 Mio/IU IFN-ß-1b (s.c.)
13	F	38	40	1	3.0	0	2.5	48	negative	IFN-ß-1a (i.m.)
14	F	22	26	1	0.0	0	0.0	48	negative	IFN-ß-1a (i.m.), GA, 9.6 Mio/IU IFN-ß-1b (s.c.)
15	F	27	35	4	4.5	**1**	4.0	48	negative	22 µg IFN-ß-1a (s.c.), IFN-ß-1a (i.m.)
16	F	25	26	2	7.0	**1**	7.0	48	negative	IFN-ß-1a (i.m.)
17	F	25	34	4	3.5	0	3.5	48	negative	44 µg IFN-ß-1a (s.c.), GA
18	M	44	50	1	6.5	0	6.5	48	negative	44 µg IFN-ß-1a (s.c.), Mitoxantrone
19	F	25	38	3	2.0	0	2.0	12	negative	22 µg IFN-ß-1a (s.c.), GA
20	F	22	28	2	2.0	0	0.0	12	negative	IFN-ß-1a (i.m.), GA
21	M	37	38	2	1.5	0	1.0	12	negative	IFN-ß-1a (i.m.)
22	F	25	26	2	2.0	0	2.0	12	negative	GA
23	F	40	51	2	4.0	0	4.0	12	negative	9.6 Mio/IU IFN-ß-1b (s.c.), GA
24	F	20	20	2	2.5	0	1.5	12	negative	IFN-ß-1a (i.m.), GA, 9.6 Mio/IU IFN-ß-1b (s.c.)
25	F	22	26	2	0.0	0	0.0	12	negative	44 µg IFN-ß-1a (s.c.), GA
26	F	21	31	3	3.5	0	3.5	12	negative	44 µg IFN-ß-1a (s.c.)

Abbreviations: EDSS, Expanded Disability Status Scale; F, female; GA, glatiramer acetate; ID, identification number IFN-ß, Interferon-beta; i.m., intramuscularly; IU, international units; M, male; NAB, natalizumab neutralizing antibodies; s.c., subcutaneously; T0, baseline; w, weeks; y, years.

1 age at which first MS-specific symptoms were reported.

2 number of relapses 12 months before natalizumab therapy.

3 number of relapses during natalizumab therapy.

4 non-persisting low titre NAB.

### Sample preparation

Peripheral venous blood was collected in commercial peripheral blood mononuclear cells (PBMC) enrichment tubes (Becton Dickinson AG, Basel, Switzerland) and processed by centrifugation at 1800 g for 20 minutes. PBMC were washed twice in PBS, resuspended in staining buffer (PBS pH 7.2, 2.5% fetal calf serum, 0.1% sodium acid) in a concentration of 1.2*10^6^ lymphocytes/ml, stained with saturating amounts of fluorescence-conjugated monoclonal antibodies for 30 minutes on ice, and fixed with 1% formaldehyde in PBS.

### Flow cytometry

Natalizumab binding (anti-human IgG4 (α-huIgG4) [clone HP6025, FITC]) and expression levels of the α_4_-integrin subunit (anti-CD49d [clones HP2/1, FITC) were investigated on four lymphocyte subpopulations (forward/sideward scatter, anti-CD3+ [clone UCHT1, ECD; identification of T cells], anti-CD19+ [clone J3-119, PC7; identification of B cells], and anti-CD16/56+ [clones 3G8 and N901, PE; identification of natural killer (NK, CD3−) and natural killer T (NKT, CD3+) cells]) by 5-color flow cytometry (Cytomics FC500, Beckman Coulter, Vienna, Austria). Isotype-matched antibodies (all IgG1) and anti-CD45+ [clone J33, FITC, PE, ECD, PC7] antibodies were used as negative and positive controls, respectively. With the exception of IgG1-FITC/PE (Exalpha, Watertown, MA, USA) all antibodies were obtained from Beckman Coulter, Vienna, Austria.

Relative fluorescence intensity (rfi) levels were calculated from median fluorescence intensities (MFI) of all leukocyte subpopulations as previously described [21], [22]. In short, rfi levels were calculated from the MFI by correcting them for the MFI of negative controls (IgG1) and relating them to the MFI of positive controls for a better inter- and intraindividual comparability. The calculation is as follows: (MFI test sample−MFI isotype control)/(MFI positive control−MFI isotype control)×1000 (annotation: multiplication by 1000 in reference to the log scale). Anti-huIgG4 rfi levels will be cited in the text as “anti-natalizumab rfi levels” and anti-CD49d rfi levels will be cited in the text as “anti-α_4_ rfi levels”.

### Determination of NAB

Determination of neutralizing anti-natalizumab antibodies in the serum was performed by ELISA at the Department of Neurology, Innsbruck Medical University, Austria [12].

### *In vitro* saturation of lymphocytes with natalizumab

Patient PBMC (1×10^6^ cells/ml) collected at baselines and after 12 weeks of natalizumab therapy were incubated with a final concentration of 10 µg/ml natalizumab. Unbound natalizumab was removed after 1 hour on ice by extensive washing. Staining and flow cytometric analysis of α_4_ integrin levels (anti-CD49d-FITC) and cell-surface bound natalizumab (anti-human IgG4-FITC) on CD3+ T cells, CD19+ B cells, NK, and NKT cells were performed as described above.

### Statistical methods

For each cell type, a repeated measures ANOVA was applied to analyze the means over time (T0, T12, T24, T36 and T48) for rfi levels of anti-hulgG4-FITC (anti-natalizumab) and anti-α_4_-FITC. Paired Fisher's least significance difference tests were used as post-hoc tests to compare means over time. To compare the cell types against each other at a fixed time point, again repeated measures ANOVA with paired Fisher's least significance difference test as post-hoc tests were applied. Whisker plots with 95% confidence intervals for the means were used to illustrate the results.

Linear regression analyses with corresponding Pearson correlation coefficients were performed with all cell types and done to compare anti-huIgG4-FITC (anti-natalizumab) rfi levels from natalizumab saturated cells collected at baseline or from cells collected during natalizumab therapy with anti-α_4_-FITC rfi levels from cells collected at baseline.

A p-value less than 5% indicates a statistically significant difference or relation. All computations and illustrations were done using Microsoft Excel (Microsoft Office 2007, Redmond, USA) and STATISTICA 10 (Hill, T. & Lewicki, P. (2007). STATISTICS: Methods and Applications. StatSoft, Tulsa, OK).

## Results

### Analysis of natalizumab binding to lymphocytes

This study comprises flow cytometric data from 26 RRMS patients who cumulatively received 320 infusions and were on 1209.9 weeks of natalizumab therapy. In detail (Table 2), 10 patients on about 1.5 years of therapy (mean 75.0 weeks, range 67.9–82.1) received 19.0±0.5 infusions with a mean infusion interval of 4.2±0.4 weeks. Eight patients on about 1 year of natalizumab therapy (mean 50.6 weeks, range 44.2–56.2) received 12.8±1.3 infusions every 4.6±0.4 weeks and another 8 patients on about 3 months of therapy (mean 12.0 weeks, range 9.2–14.8) received mean 3.8±0.5 infusions every 4.5±0.5 weeks.

**Table 2:** Patient Characteristics - Means, Medians, and Standard Deviations of Treatment Parameters.

		Number of Infusion		Weeks on Therapy		Infusion Interval (weeks)	
Duration of Therapy (approx.)	Number of Patients	mean (SD)	median (SD)	mean (SD)	median (SD)	mean (SD)	median (SD)
72 weeks	10	19.0 (0.5)	19.0 (0.5)	75.0 (7.1)	77.1 (7.1)	4.2 (0.4)	4.3 (0.4)
48 weeks	8	12.8 (1.3)	13.0 (1.3)	50.6 (6.2)	52.3 (6.2)	4.6 (0.4)	4.4 (0.4)
12 weeks	8	3.8 (0.5)	4.0 (0.5)	12.0 (2.8)	12.4 (2.8)	4.5 (0.5)	4.2 (0.5)

Abbreviations: approx., approximately; SD, standard deviation.

Natalizumab binding and surface availability of the α_4_ integrin subunit on four lymphocyte subpopulations including CD3+ T cells, CD19+ B cells, NK, and NKT cells were investigated. One patient (#1) developed NAB and will be discussed separately because results differed significantly.

**Figure 1:**
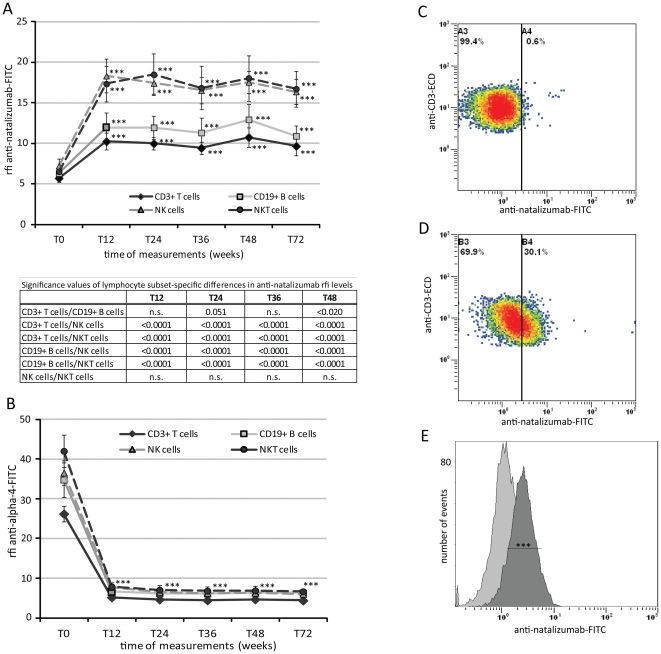
Flow cytometric analysis of natalizumab binding to four different lymphocyte subsets from RRMS patients under therapy. Increase of anti-natalizumab rfi levels (A) and decrease of anti-α_4_ rfi levels (B) illustrated for CD3+ T cells (filled diamond), CD19+ B cells (open square), NK cells (open triangle), and NKT cells (filled circle) at weeks 12 (T12, n = 17), 24 (T24, n = 17), 36 (T36, n = 17), 48 (T48, n = 17), and 72 (T72, n = 10) compared to background (anti-natalizumab) respectively baseline (anti-α_4_ rfi) levels (T0, n = 17). Significance levels calculated for lymphocyte subset-specific differences of anti-natalizumab rfi levels at each measurement during therapy are summarized in the associated table. Representative FACS plots show anti-natalizumab rfi levels on CD3+ T cells at baseline, i.e. background signaling, (C) and after 12 weeks of natalizumab therapy (D). Anti-natalizumab median fluorescence intensities of the above two FACS plots depicted as overlapping histrograms (E). ECD, electron coupled dye; FITC, Fluorescein isothiocyanate; NK, natural killer cells; NKT, natural killer T cells; n.s., non significant; Rfi, relative fluorescence intensity; T(x), Time of measurement (weeks); triple stars (***) highlight highly significant results (p<0.001); vertical bars represent 95% confidential intervals.

Anti-natalizumab rfi levels on PBMC from 17 patients (#2–18) were measured in 12 weeks intervals from baseline until up to 72 weeks of natalizumab therapy. After 12 weeks, we observed a significant increase in mean anti-natalizumab rfi levels (p<0.001) on all lymphocyte subsets compared to background levels obtained at baseline. The increase was least in CD3+ T cells (1.8±0.5 fold) and most pronounced in NK/NKT cells (2.7±0.9/0.8 fold) (Figure 1A). Increases in anti-natalizumab rfi levels were accompanied by a sharp decline of about 80% in mean α_4_ rfi levels (p<0.001, Figure 1B). Both, the increase in anti-natalizumab rfi levels, and as shown before [23], the decrease in anti-α_4_ rfi levels were stable from the week 12 through the week 72 measurements.

Anti-natalizumab rfi levels of PBMC from RRMS patients collected at baseline were comparable to those of PBMC from healthy controls and represent background signals (data not shown).

We found that natalizumab binding to NK cells and NKT cells was significantly higher than natalizumab binding to CD3+ T cells (p<0.001, both NK and NKT cells) and CD19+ B cells (p<0.001, both NK and NKT cells). B cells had slightly higher anti-natalizumab rfi levels than T cells. Natalizumab binding to NK cells and NKT cells did not differ significantly (Figure 1A).

A potential unspecific adsorption of natalizumab to low-affinity IgG Fc receptors (CD16) on NK cells was excluded by analyzing natalizumab binding after *in vitro* treatment of cells with saturating amounts of natalizumab in the presence and absence of Fc receptor saturation reagent (data not shown).

### Natalizumab therapy and the composition of lymphocyte subsets

We examined relative frequencies of circulating lymphocytes which showed significant and persistent increases of CD19+ B cells (p<0.001) in patients on natalizumab therapy and expansions of NK cells which were significant at weeks 36 (p<0.001), 48 (p<0.01), and 72 (p<0.01). Mean frequencies of the CD3+ T cell population were significantly reduced (p<0.001) and mean frequencies of NKT cells were largely unchanged (Table 3).

**Table 3:** Changes in Frequencies of Lymphocyte Subsets during Natalizumab Therapy.

	baseline	12 weeks	24 weeks	36 weeks	48 weeks	72 weeks
	% (SD)	% (SD)	% (SD)	% (SD)	% (SD)	% (SD)
**CD3+ T cells**	74.8 (6.2)	65.3 (5.9)***	65.1 (5.3)***	62.0 (8.2)***	62.9 (5.2)***	63,7 (6.0)***
**CD19+ B cells**	9.1 (3.5)	17.3 (6,6)***	17.9 (6.3)***	19.1 (8.0)***	18.8 (6.7)***	17.5 (3.8)***
**NK cells**	11.1 (4.7)	12.5 (3.7)	12.6 (4.6)	14.5 (4.3)***	13.6 (3.5)**	14.4 (4.3)**
**NKT cells**	5.0 (2.7)	4.6 (2.7)	4.4 (2.5)	4.4 (2.4)*	4.7 (2.6)	4.,4 (2.7)

Abbreviations: NK, natural killer; NKT, natural killer T cells; SD, standard deviation;

*) p<0.05;

**) p<0.01;

***) <0.001.

Single patients showed strongly deviating cell frequencies of NK cells. At baseline, NK cell frequencies were between 6.9% to 14.3% in 15 patients but 21.3% and 22.1% in 2 patients (#10 and #11). In contrast to the overall trend, NK cell frequencies of these 2 patients decreased. One of them (#10) reported disease activity.

### Cell-bound Natalizumab and α_4_-integrin baseline levels – two correlations by comparison

**Figure 2:**
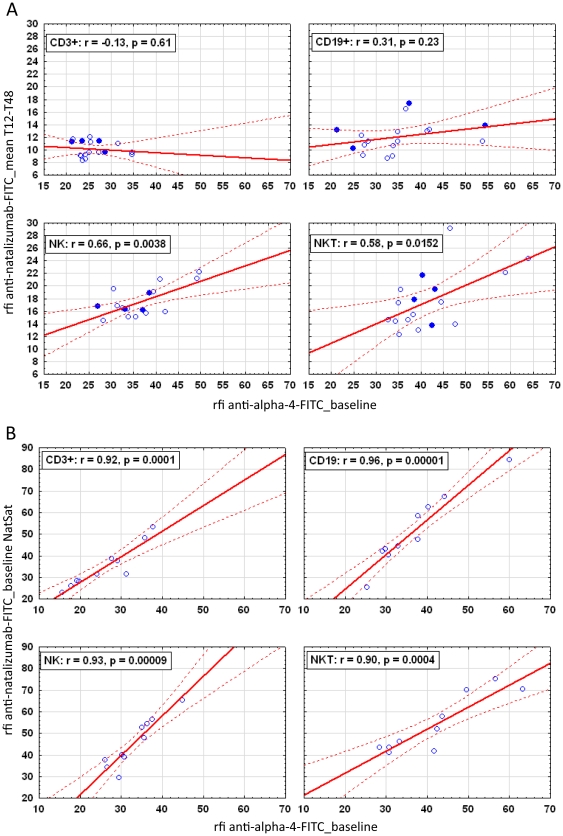
Correlation results between cell-bound natalizumab and α_4_ integrin surface levels. (A) Anti-natalizumab rfi levels of week 12–48 measurements correlated with anti-α_4_ rfi levels from cells collected at baseline (n = 17). Filled symbols highlight patients who reported disease activity. (B) Anti-natalizumab rfi levels from *in vitro* saturated cells collected at baseline correlated with anti-α_4_ rfi levels from natalizumab-naïve cells of the same blood collection (n = 10). FITC, Fluorescein isothiocyanate; NatSat, *in vitro* treated cells with saturating amounts of natalizumab; NK, natural killer cells; NKT, natural killer T cells; rfi, relative fluorescence intensity; T12–48, time of measurements at weeks 12–48.

From the simple context that natalizumab blocks the α_4_-integrin, a direct-proportional relation between lymphocyte subset-specific differences in natalizumab binding and surface expression of α_4_-integrin appeared conclusive. Accordingly, we correlated anti-natalizumab rfi levels of lymphocytes collected at weeks 12, 24, 36, and 48 (patients #2–18) with the anti-α_4_ levels of natalizumab-naïve lymphocytes collected at baseline (Figure 2A). Mean rfi levels of anti-natalizumab positively correlated with anti-α_4_ baseline levels in NK (r = 0.66, p = 0.0038) and NKT cells (r = 0.58, p = 0.015) but not with CD3+ T cells (r = −0.13, p = 0.61) nor with CD19+ B cells (r = 0.31, p = 0.23).

This poor overall correlation was not expected. In a second approach we used treatment-naive PBMC from 10 patients collected at baseline and correlated anti-natalizumab rfi levels of *in vitro* saturated lymphocytes with anti-α_4_ integrin levels of natalizumab-naïve lymphocytes (Figure 2B). The results showed highly significant and strongly positive correlations in all subsets: CD3+ T cells (r = 0.92, p = 0.0001), CD19+ B cells (r = 0.96, p = 0.00001), NK cells (r = 0.93, p = 0.00009), and NKT cells (r = 0.90, p = 0.0004). Thus, the association of lymphocyte subset-specific differences in α_4_-integrin expression levels and detectability of natalizumab was established.

### Natalizumab saturation and natalizumab binding capacity

**Figure 3:**
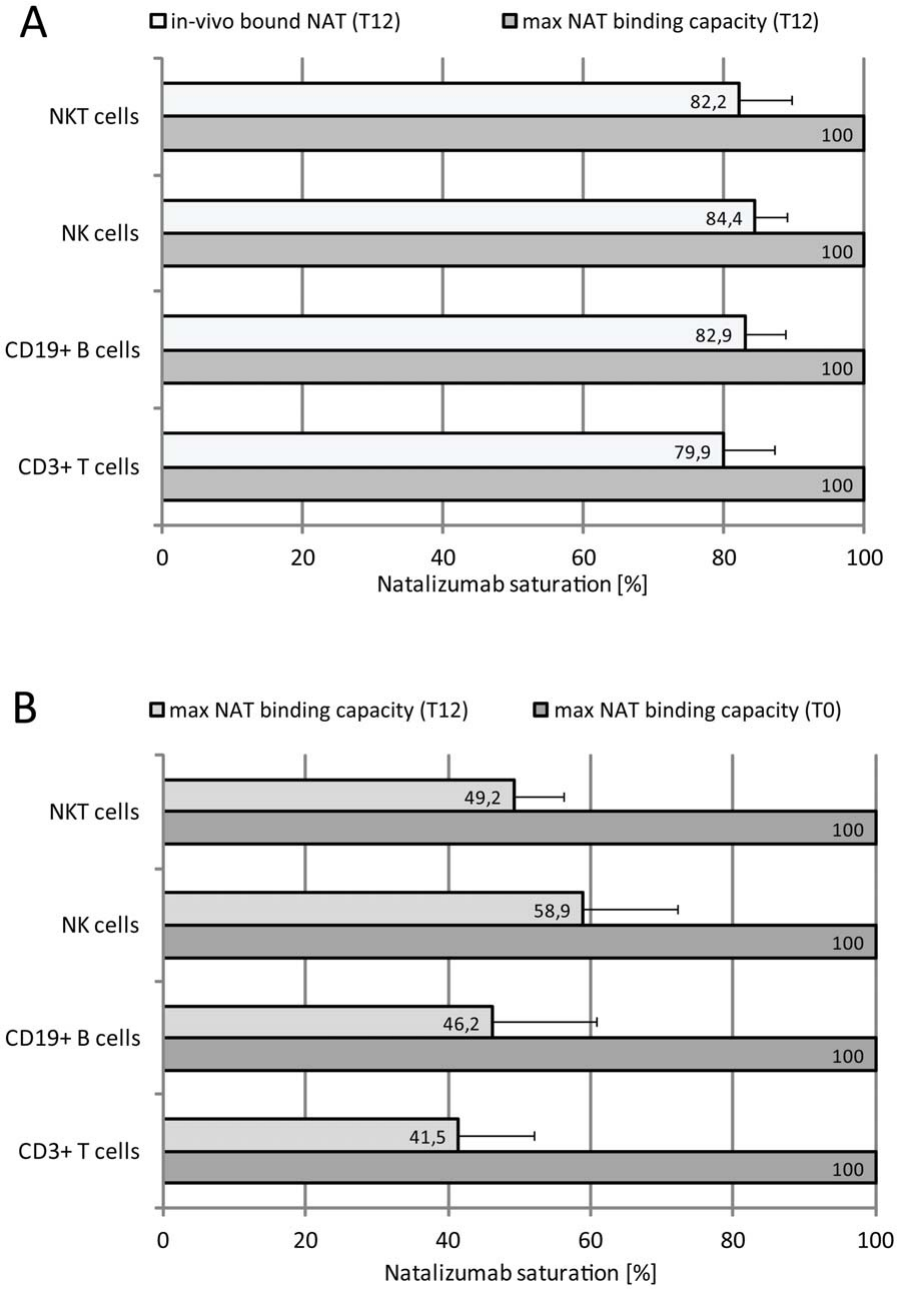
Maximum natalizumab binding capacity and natalizumab saturation before and during natalizumab therapy. (A) Percent natalizumab saturation of T cells, B cells, NK cells, and NKT cells collected at week 12 (T12, n = 8) during therapy related to maximum natalizumab binding capacity of lymphocytes from the same blood collection after *in vitro* treatment with saturating amount of natalizumab (100%). (B) Percent maximum natalizumab binding capacity of *in vitro* saturated T cells, B cells, NK cells, and NKT cells collected at week 12 (T12, n = 8) related to maximum natalizumab binding capacity of *in vitro* saturated lymphocytes from the same donors collected at baseline (100%). Vertical bars represent 95% confidential intervals. Max, maximum; NAT, natalizumab; NK, natural killer cells; NKT, natural killer T cells; T(x), time of measurement (weeks).

One possible explanation for the differing correlation results between *in vitro* saturated lymphocytes collected at baseline and lymphocytes with *in vivo* bound natalizumab collected during therapy was, that latter lacked saturation. This led us to investigate saturation levels of lymphocytes from patients under natalizumab therapy. PBMC from 8 patients collected at baseline and at week 12 were *in vitro* treated with saturating amounts of natalizumab or buffer and analyzed by flow cytometry. We found that additional *in vitro* treatment with natalizumab only caused a minor increase of anti-natalizumab rfi levels compared to therapy-derived *in vivo* natalizumab levels which was 1.3±0.2 fold in case of CD3+ T cells, and 1.2±0.1 fold for CD19+ B cells, NK cells, and NKT cells. With the maximum natalizumab binding capacity of *in vitro* saturated lymphocytes defined as 100% saturation, we showed that lymphocytes collected at week 12 were approximately 80% saturated (Figure 3A). Thus, we excluded lack of natalizumab as cause for the poor correlation results. Next, we compared the maximum natalizumab binding capacities of *in vitro* saturated lymphocytes collected at baseline and *in vitro* saturated lymphocytes collected during therapy. We noticed significantly higher anti-natalizumab rfi levels on *in vitro* saturated lymphocytes collected at baseline compared to *in vitro* saturated lymphocytes collected during therapy (p<0.01). The fold increase in anti-natalizumab rfi levels in relation to background signals of lymphocytes collected at baseline was 5.9±1.5 in CD3+ T cells, 5.6±1.5 in CD19+ B cells, 6.1±2.0 in NK cells, and 7.7±2.5 in NK cells. This was 2–3 times the anti-natalizumab rfi values of *in vitro* saturated cells collected under therapy. With anti-natalizumab rfi levels of *in vitro* saturated lymphocytes collected at baseline defined as 100% natalizumab binding capacity revealed a strongly diminished natalizumab binding capacity of *in vitro* saturated lymphocytes collected under therapy which was 58.9%±19.1 in NK cells, 49.2%±10.0 in NKT cells, 46.2%±21.1 in CD19+ B cells, and 41.1%±15.4 in CD3+ T cells (Figure 3B).

Apparently, lymphocytes collected during therapy have a reduced capacity to bind natalizumab compared to treatment-naive lymphocytes. A therapy-induced reduction in the maximum natalizumab binding capacity of cells is a plausible explanation for the poor correlation in figure 2A.

### Natalizumab accumulations on lymphocytes from individual patients

**Figure 4:**
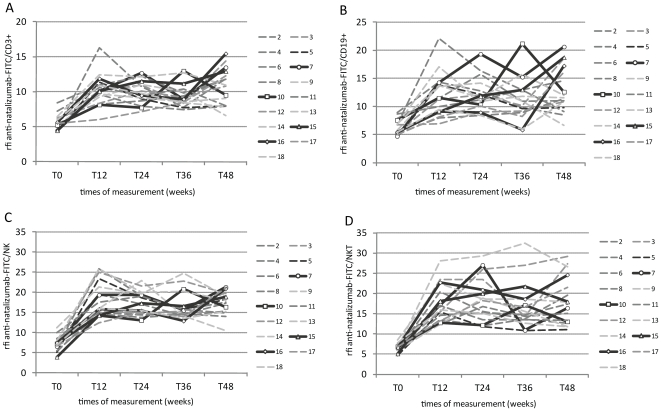
Individual courses of natalizumab binding during therapy. Anti-natalizumab rfi of each patient and measurement depicted for CD3+ T cells (A), for CD19+ B cells (B), for NK cells(C), for NKT cells(D). Graphs of patients with reported disease activity are highlighted as bold lines with open circles, squares, triangles, or diamonds as identification marks. FITC, Fluorescein isothiocyanate; NK, natural killer cells; NKT, natural killer T cells; Rfi, relative fluorescence intensity; Tx, time of measurement (weeks).

Figure 4 summarizes all measurements from patients #2–18 from baseline until week 48 in four charts, one for each lymphocyte subset. Graphs #7, #10, #15 and #16 are bold lines with identification marks and highlight the four patients who reported disease activity during natalizumab therapy. The graphs revealed pronounced inter- but also intra-individual differences in natalizumab binding mainly as temporary increases (peaks). To quantify these differences, rfi levels were translated into fold increases by dividing anti-natalizumab rfi levels of the week 12, 24, 36, and 48 measurements by the anti-natalizumab background rfi levels. A lymphocyte subset-specific cut-off was determined by adding the mean fold increase in anti-natalizumab rfi levels of weeks 12 to 48 from patients #2–18 and one standard deviation. With this cut-off significant peaks of natalizumab accumulations on lymphocytes were evaluated in the context of disease activity at weeks 12, 24, 36, and 48 (Table 4).

**Table 4:** Evaluation of natalizumab accumulations on lymphocyte subsets from individual patients at single time-points.

	CD3+ T cells				CD19+ B cells				NK cells				NKT cells			
ID	T12/T0	T24/T0	T36/T0	T48/T0	T12/T0	T24/T0	T36/T0	T48/T0	T12/T0	T24/T0	T36/T0	T48/T0	T12/T0	T24/T0	T36/T0	T48/T0
2	1.65	1.37	1.28	1.37	1.60	1.43	1.10	0.95	2.56	2.23	1.86	2.02	2.16	1.92	2.17	2.02
3	1.79	1.92	1.47	**2.52**	2.12	2.26	1.91	**3.48**	2.94	2.80	2.92	**3.78**	**3.88**	**3.88**	2.37	**4.52**
4	**3.37**	2.22	1.61	**2.46**	**3.24**	2.40	1.90	2.14	**4.09**	3.03	2.49	2.77	3.36	2.56	2.26	3.22
5	**2.57**	2.05	1.76	1.81	**3.03**	2.67	2.10	2.15	**4.55**	**3.62**	3.06	**3.55**	3.37	2.57	2.37	2.42
6	1.53	1.63	1.76	2.20	1.55	1.62	1.74	2.20	2.66	2.51	2.64	3.17	2.43	2.55	2.47	2.90
**7**	2.27	**2.85+**	2.01	**3.03+**	**3.04+**	**4.12+**	**3.25+**	**4.40+**	2.94	2.93	2.36	3.24	2.69	**4.23+**	1.70	2.57
8	1.29	1.45	1.16	1.40	1.45	2.00	1.39	1.40	1.76	1.91	1.58	1.49	1.80	2.44	2.09	2.19
9	1.75	2.08	1.89	1.24	2.35	2.44	1.89	1.14	1.92	2.75	1.70	1.26	2.09	3.19	2.64	2.00
**10**	1.55	1.47	**2.48+**	1.82	1.52	1.36	**2.80+**	1.67	1.97	1.80	2.86	2.25	1.86	1.77	2.49	1.90
11	1.46	1.31	1.44	1.11	1.14	1.29	1.50	1.05	1.75	1.68	1.70	1.63	2.23	1.78	1.78	1.75
12	1.34	1.62	1.47	1.72	1.02	1.33	1.36	1.61	3.15	2.72	2.87	2.48	2.23	3.75	**3.90**	**4.21**
13	1.93	1.89	1.97	1.70	1.99	1.68	2.28	1.74	1.89	1.74	2.20	1.70	**4.31**	**4.50**	**4.99**	**4.08**
14	1.61	1.52	1.43	1.51	2.51	1.62	1.74	1.69	3.28	2.53	2.56	2.28	2.65	2.20	2.20	2.11
**15**	2.26	**2.58+**	**2.51+**	**2.90+**	1.66	2.17	2.38	**3.43+**	**3.61+**	**4.37+**	**4.20+**	**4.79+**	**3.56+**	**3.93+**	**4.27+**	3.53
**16**	2.11	1.68	1.58	**2.74+**	1.67	1.64	1.07	**3.16+**	2.12	2.07	1.73	2.81	3.44	3.17	2.82	**3.73+**
17	1.10	1.31	1.55	2.25	1.64	2.03	1.66	**3.25**	1.87	2.24	2.11	3.16	1.96	2.68	2.45	3.18
18	1.83	1.64	1.41	1.32	1.96	1.98	1.83	2.20	2.29	2.23	1.95	2.19	2.04	1.93	1.62	1.50
**mean**					**1.82**			**2.02**			**2.56**			**2.76**		
SD					0.50			0.74			0.78			0.87		
**Cut-off** 1					**2.32**			**2.76**			**3.34**			**3.63**		

Abbreviations: ID, identification number; NK, natural killer cells; NKT, natural killer T cells; SD, standard deviation; T, time of measurement; Tx/T0, quotient of anti-natalizumab rfi levels of measurements performed during natalizumab therapy (T12,…,48) and background rfi levels from measurements performed at baseline (T0);

Bold ID numbers highlight patients with reported disease activity; bold results highlight values above cut-off; bold results with plus signs highlight values above cut-off of patients with reported disease activity.

1 cut-off defined as sum of means plus one standard deviation.

Seven out of 17 patients had anti-natalizumab rfi levels above cut-off at least once on CD3+ T cells, 8/17 on CD19+ B cells, 4/17 on NK cells, and 6/17 on NKT cells. All four patients with reported disease activity showed peaks in the CD3+ T (4/7) and CD19+ B (4/8) lymphocyte subsets. In case of NK and NKT cells only 1/4 and 2/6 were patients with reported disease activity.

Intraindividual differences in natalizumab binding either relate to peaks at discrete measurements or to increased anti-natalizumab rfi levels over minimum three time-points which are primarily restricted to one lymphocyte subset. Patient #7, for example, had pronounced anti-natalizumab rfi levels mainly on CD19+ B cells, patient #5 on NK cells, patients #3 and #13 on NKT cells, and patient #15 had them on CD3+ T cells, NK, and NKT cells. Peaks in cell-bound natalizumab could not be associated with higher α_4_ integrin baseline levels: the range of α_4_ baseline rfi levels as defined by all patients was 21.2–34.6 on CD3+ T cells, 21.3–54.3 on CD19+ B cells, 27.0–49.6 on NK cells, and 32.7–64.1 on NKT cells. The baseline α_4_ rfi levels of patient #15, for example, were within the patient-defined ranges (23.5 on T cells, 27.0 on NK, and 43.1 on NKT cells). Similar was the case with α_4_ baseline rfi levels on B cells from patient #7 (37.5), on NK cells from patient #5 (39.5), and on NKT cells from patients #3 (58.9) and #13 (46.5). These results illustrate that natalizumab accumulations are not dependent on baseline α_4_ levels.

### The case of a patient with non-persistent NAB

**Figure 5:**
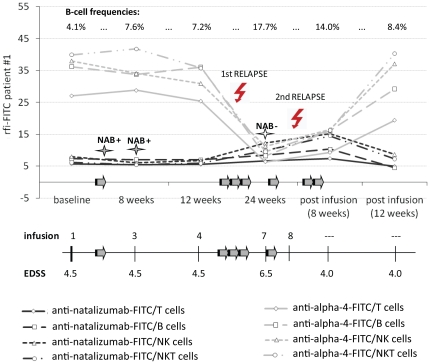
Flow cytometry in a case with natalizumab neutralizing antibodies (NAB). Anti-natalizumab-FITC rfi levels (black lines) and anti-α_4_-FITC rfi levels (grey lines) on CD3+ T cells (solid line), CD19+ B cells (dashed line), NK cells (dotted line), and NKT cells (dot and dash line) from one patient with non-persistent NAB. The numbers of infusions and EDSS scores are specified in a separate diagram. Each bold arrow plotted onto the x-axis represents 4 weeks. NAB were measured from sera collected at weeks 4, 8, and 24. Frequencies of the CD19+ B cell population appear in brackets [%]. EDSS, expanded disability status scale FITC, Fluorescein isothiocyanate; NAB, natalizumab neutralizing antibodies; NK, natural killer cells; NKT, natural killer T cells; rfi, relative fluorescence intensity.

Flow cytometric analysis of immune cells from patient #1 revealed unaltered high α_4_ integrin levels and a lacking increase in anti-natalizumab rfi levels at the week 12 measurement despite the second and third infusions (Figure 5). This was highly suggestive of NAB. In the course of ongoing treatment, α_4_ rfi levels declined and natalizumab could be detected. PBMC collected shortly before the seventh infusion showed an increase in anti-natalizumab rfi levels of 15.3% on T cells, 14.8% on B cells, 53.4% on NK cells, and 59.8% on NKT cells compared to background levels. This indicated that NAB were non-persistent. In routine laboratory testing the presence of low titer NAB (sera collected at weeks 4 and 8) and their resolution (serum collected at week 24) were confirmed. NAB were non-persistent, still, the patient suffered a further relapse and natalizumab therapy was terminated after the eighth infusion at week 28. Flow cytometric data collected 8 weeks after the last infusion showed a slight recovery of α_4_ integrin levels, but anti-natalizumab rfi levels were also still elevated on B cells (+40.9%), NK cells (+90.1%), NKT cells (+133.8%), and to a lesser extent on T cells (+29.3%). Twelve weeks after the last infusion anti-natalizumab rfi levels were no longer above background levels and α_4_ integrin rfi levels approached baseline levels again.

The B cell population showed a slight elevation from 4.1% at baseline to 7.6% and 7.2% at the weeks 8 and 12 measurements despite the presence of NAB. As soon as NAB resolved the B cell population increased to 17.7% at week 24. Eight and twelve weeks after the last infusion B cell numbers slowly declined along with the wash-out of natalizumab to 14.0% and 8.4%, respectively.

## Discussion

Natalizumab is a highly effective drug for the treatment of RRMS [18]. The four-week intravenous application regimen requires admittance to a medical center and facilitates exact reports on the patients' adherence to infusion intervals which is important for molecular studies like this. Accordingly, mean and median duration of therapy, numbers of infusions, and infusion intervals have been assessed from all patients included in this study. Our goal was to determine if serial analysis of natalizumab binding to lymphocytes from RRMS patients by flow cytometry has the potential to be applied as a biomarker for the individual treatment response.

First, we determined that flow cytometry and the use of a fluorescence-labeled monoclonal antibody specifically recognizing the human IgG4 framework of natalizumab are appropriate to investigate *in vivo* bound natalizumab on immune cells *ex vivo*. We show here that standard 300 mg natalizumab infusions induced a significant increase of mean anti-natalizumab rfi levels on lymphocytes compared to baseline which was most prominent on NK and NKT cells (2.7 fold at the 12 weeks measurement) and least pronounced in T cells (1.8 fold at the 12 weeks measurement). The increase was consistent across the observation period until up to 72 weeks of therapy. A concurrent sharp decline of approximately 80% in mean α_4_ integrin levels confirmed specificity of results because cell-bound natalizumab interfered with attachment of our anti-α_4_ detection antibody (clone HP2/1).

Examination of the course of natalizumab binding to lymphocytes from individual patients, however, showed pronounced intraindividual differences which appeared as natalizumab accumulations at single measurements. To discriminate method-based fluctuations from significant peaks a cut-off was determined using mean anti-natalizumab rfi levels plus one standard deviation. With this cut-off we singled out patients with strikingly high natalizumab binding to one or more lymphocyte subsets and found this to be the case in about half of the patients in T and B cells, and about 25% of patients in NK and NKT cells. Excitingly, all of the four patients with reported clinical disease activity had natalizumab accumulations in T and B cells. This finding provides evidence that flow cytometric analysis of natalizumab binding to T and B lymphocytes actually deserves attention as a potential biomarker for disease activity. The occurrence of natalizumab peaks in some other patients fits perfectly to the fact that immunological or MRI disease activities are only partly represented by clinical manifestations.

The pivotal question certainly is - what are the underlying mechanisms? An approach to this question requires knowledge on natalizumab effects beyond the postulated blockade of the α_4_ integrin.

Natalizumab binding was most pronounced on NK and NKT cells which suggested a higher α_4_ integrin surface expression on these two lymphocyte subsets. We confirmed this by demonstrating a direct dependency and strong correlation between α_4_ integrin levels and the extent of natalizumab binding after *in vitro* saturation on lymphocytes from untreated MS patients. Importantly, the positive correlation was lost in T cells and B cells when comparing α_4_ integrin levels of treatment-naive cells collected at baseline with natalizumab binding to lymphocytes collected after 12 weeks of therapy. And, the positive correlation was still existent but less significant in NK and NKT cells. The loss of correlation indicates that natalizumab therapy leads to altered α_4_ integrin surface levels and that T and B cells apparently are more affected than NK and NKT cells. This observation is in line with Niino et al [24] who reported that natalizumab therapy induced a down-regulation of α_4_ integrin expression on immune cells. In contrast to us, they investigated α_4_ integrin levels using a detection antibody of different epitope specificity than natalizumab. We were less interested in the differential surface expressions of α_4_ integrin but wanted to investigate the impact of natalizumab therapy on the overall capacity of cells to bind natalizumab. Accordingly, we analyzed the maximum natalizumab binding capacity after *in vitro* saturation of lymphocytes from patients collected at baseline and after three months of therapy. We found an impressive reduction of about 40% (NK cells) to about 60% (T cells) in the maximum natalizumab binding capacity of lymphocytes from patients on therapy compared to lymphocytes from the same patients prior to treatment. Again, T and B cells showed a more pronounced effect and higher reduction in their natalizumab binding capacity than the two other subsets.

These findings led us to suggest that natalizumab primarily affects T and B cells via lower abundance in α_4_ integrin surface levels. Specifically responsive to therapy, these cells also might be exceptionally sensitive and reactive in the context of immune activities evidenced as accumulations of natalizumab. The more so as the antiviral cytokine interferon-alpha, for example, has been shown to up-regulate surface expression of the α_4_ integrin on T cells [25]. This could explain both that natalizumab peaks above cut-off occurred in all patients with reported clinical disease activity and that they were more frequent in T and B cells compared to NK and NKT cells.

NK and NKT cells have considerably higher α_4_ integrin levels than T and B cells, but the relevance is controversial. Higher α_4_ integrin levels on CD8+ T cells, for example, have been implicated in the altered CD4+/CD8+ T cell ratio in the cerebrospinal fluid of natalizumab-treated patients [26]. Others have shown that α_4_ integrin levels do not necessarily correlate with the adhesive property of cells [27]. Generally, the role of NK and NKT cells in the pathogenesis of MS is not resolved. NK cells have been reported to exert a decreased cytotoxic activity during relapses [28], [29], to interfere with autoimmune responses by secreting anti-inflammatory cytokines [30], and to be involved in the decrease of relapse rates during pregnancy [31]. Moreover, NK cells have been shown to expand in response to MS therapeutics, including interferon-beta [32], the monoclonal antibody daclizumab [33], and, very recently, also natalizumab [11]. We too, observed an increase of NK cell numbers during natalizumab therapy in all but two of our patients. Latter had striking high NK cell frequencies at baseline which actually decrease in the course of therapy. Natalizumab apparently exerts differential effects on NK cell subsets besides the supposed boosting of immune regulatory NK cells. Future studies on NK cells classified as regulatory or cytotoxic subsets, activators or inhibitors of target cell killing, will provide substantial insights into the interactions between natalizumab and NK cells, and will possibly answer the question whether natalizumab binding to NK cells rather interferes with their immune-modulatory function or with the immune pathogenesis of MS.

A hot topic emerged from the major decrease in maximum natalizumab binding capacity of lymphocytes during natalizumab treatment. It involves the rationale for monthly applications of 300 mg natalizumab and the feasibility of patient-tailored infusion intervals with regards to opportunistic side effects like PML. Early safety and pharmacokinetic studies reported natalizumab serum concentrations detectable until 3–8 weeks after a single 3 mg/kg intravenous dose [34]. Elimination of biologics like monoclonal antibodies is generally slow and involves proteolysis, renal filtration, and intracellular catabolism after endocytosis [35]. Salvage from lysosomal degradation by Fc receptors of neonatal (FcRn) and escape from immune-mediated elimination mechanisms due to its IgG4 framework cause the extremely low systemic clearance and prolonged terminal half-life of natalizumab [6], [36]. Rispens et al [37] recently developed a sophisticated ELISA for quantifying serum natalizumab and identified single patients with exceptional high natalizumab levels four weeks post infusion. The substantial differences in individual pharmacokinetics and the reduced cellular natalizumab surface load during therapy add one more indication for monitoring natalizumab binding to immune cells by flow cytometry. The possibilities and procedures for implementing patient-optimized infusion intervals should be investigated in controlled studies.

Last, we discuss the issue of monitoring the occurrence of NAB by flow cytometry. Despite the fact of an increase in allergic reactions, Calabresi et al. [18] suggested not to test for NAB within the first months of therapy to avoid uncertainties regarding treatment continuation in case of positive results. Their indication for NAB testing is restricted to identify patients with persistent NAB which are associated with a continuing insufficient treatment response. In contrast, Defer et al. [38] recently suggested to investigate surface expression of the α_4_ integrin by flow cytometry as a surrogate marker for treatment efficacy. In our patient occurrence of NAB was clearly evidenced by lack of anti-natalizumab rfi levels and unchanged baseline α_4_ rfi levels despite repeated infusions. NAB were non-persistent as indicated by a decline in α_4_ surface levels prior to the seventh infusion. Nonetheless, the patient suffered a second relapse resulting in the termination of natalizumab therapy. The patient showed a very slow recovery of α_4_ levels which, on T cells, were not back at baseline even 12 weeks after the last infusion.

A novel observation is that assessment of B cell frequencies apparently functions as sensitive marker for residual natalizumab activity which is not detected by flow cytometric analysis of α_4_ integrin or anti-natalizumab levels. B cell frequencies doubled with start of therapy despite presence of NAB, quadruplicated upon resolution of NAB, and were still twice the baseline levels at the final measurement 12 weeks after the last infusion. Our case reveals that repeated immune cell monitoring by flow cytometry is feasible and indicative. It provides immediate information on persistence or non-persistence of NAB which can be continuously matched with clinical parameters and the patient's condition. Such close observations of patients with NAB will increase experience from individual disease courses and provide a basis for more precise case-to-case specific decisions for affected individuals.

Summing up, clinical effectiveness of natalizumab is tightly linked to blocking the α_4_ integrin and can be easily investigated by flow cytometry. In this study we observed significant accumulations of natalizumab on patient lymphocytes that possibly signalize immunologic disease activity. We found an expansion of NK cells in most patients and a reduction in two individuals which could indicate further unknown effects of natalizumab. We saw a strong decrease in the natalizumab binding capacity of lymphocytes during therapy which suggests a certain potential to customize infusion intervals.

We concede that the small number of patients with relapse activity and NAB presented in this pilot study only allow careful interpretations. Nevertheless, investigating natalizumab binding to lymphocytes adds important information for the clinician. Flow cytometric analysis of cell-bound natalizumab appears promising in the search for biomarkers for treatment effect and pending relapse activity in individual cases. And, it might turn out the basis for further studies on optimizing infusion intervals which would greatly improve treatment safety.
